# Functional whole-brain mechanisms underlying effects of tDCS on athletic performance of male rowing athletes revealed by resting-state fMRI

**DOI:** 10.3389/fpsyg.2022.1002548

**Published:** 2022-10-04

**Authors:** Ming Ma, Yan Xu, Ziliang Xiang, Xi Yang, Jianye Guo, Yong Zhao, Zhenghua Hou, Yuxu Feng, Jianhuai Chen, Yonggui Yuan

**Affiliations:** ^1^Department of Rehabilitation, Zhongda Hospital, School of Medicine, Southeast University, Nanjing, China; ^2^Department of Andrology, Jiangsu Province Hospital of Chinese Medicine, Affiliated Hospital of Nanjing University of Chinese Medicine, Nanjing, China; ^3^Department of Psychosomatics and Psychiatry, Zhongda Hospital, School of Medicine, Southeast University, Nanjing, China; ^4^Department of Orthopaedics, Pukou Central Hospital, PuKou Branch Hospital of Jiangsu Province Hospital, Nanjing, China

**Keywords:** transcranial direct current stimulation, resting-state functional magnetic resonance imaging, amplitude of low-frequency fluctuation, regional homogeneity, graph theory analysis, athletic performance

## Abstract

**Introduction:**

Transcranial direct current stimulation (tDCS) is a noninvasive brain stimulation technique that applied to modulate brain activity and enhance motor recovery. However, the neurobiological substrates underlying the effects of tDCS on brain function remain poorly understood. This study aimed to investigate the central mechanisms of tDCS on improving the athletic performance of male rowing athletes.

**Methods:**

Twelve right-handed male professional rowing athletes received tDCS over the left primary motor cortex while undergoing regular training. The resting-state functional magnetic resonance imaging (rs-fMRI) data were acquired before and after tDCS. Measures of amplitude of low-frequency fluctuation (ALFF) and regional homogeneity (ReHo) were calculated and compared between baseline and follow-up, as well as topological measures including global and local efficiency of functional brain networks constructed by graph theoretical analysis.

**Results:**

Male rowing athletes showed increased isokinetic muscle strength of the left knee and left shoulder after tDCS. Increased ALFF values were found in the right precentral gyrus of male rowing athletes after tDCS when compared with those before tDCS. In addition, male rowing athletes showed increased ReHo values in the left paracentral lobule following tDCS. Moreover, increased nodal global efficiency was identified in the left inferior frontal gyrus (opercular part) of male rowing athletes after tDCS.

**Conclusion:**

The findings suggested that simultaneous tDCS-induced excitation over the primary motor cortex might potentially improve the overall athletic performance in male rowing athletes through the right precentral gyrus and left paracentral lobule, as well as left inferior frontal gyrus.

## Introduction

The human brain has a remarkable degree of structural and functional plasticity in response to learnings, training and experiences including sensory and motor experiences, even after a relatively short time of training (Dayan and Cohen, 2011; Huang et al., 2018). Previous neuroimaging studies of professional athletic training had demonstrated longitudinal structural and functional changes of the brain following exposure to training, which suggested that the architecture of human brain could be reshaped by motor skill training (Wang et al., 2016; Yamazaki et al., 2019). Expert athletes had extraordinary motor skills than non-experts and different brain activation were found between experts and novices, which were considered to be related with motor planning and motor performance (Wright et al., 2013; Debarnot et al., 2014; Moscatelli et al., 2016). The posterior cingulate, amygdala and basal ganglia were active only in novice golfers, whereas the activate brain regions were primarily located in the superior parietal lobule, dorsal lateral premotor area and occipital area of expert golfers, which suggested that the differences of motor performance between experts and novices were associated with the organization of functional brain networks during motor planning (Milton et al., 2007). In addition, the anterior cingulate, temporal and occipital gyrus were activated when experts were aiming, whereas the frontal area and posterior cingulate gyrus were activated when novices were aiming, which suggested that expert and novice archers showed differences in the neural networks (Kim et al., 2008). Therefore, extensive practice might lead to a focused and efficient organization of brain network for experts, whereas the less efficient brain network of novices might have difficulty in filtering out irrelevant information.

Transcranial direct current stimulation (tDCS) is a non-invasive brain stimulation technique, which has been applied to improve mental and physical performance in sports through modulating cortical excitability involved in physiological and cognitive reactions (Foerster et al., 2013; Nikooharf Salehi et al., 2022). In recent years, there has been an increasing interest in the studies of using non-pharmacological brain stimulation approaches to enhance athletic performance and researchers have addressed the effects of tDCS on athletic performance, which might be attributed to altered spontaneous neural activity in the brain (Machado et al., 2019; Shyamali Kaushalya et al., 2022). The primary motor cortex (M1) was the main target brain region of tDCS (Lerma-Lara et al., 2021; Parma et al., 2021). Simultaneous stimulation of the primary motor cortex and spinal cord has been found to be effective in improving the athletic and cognitive performance of experienced boxers through neuromodulation, which may influence the success of athletes in the intense professional competitions (Kamali et al., 2021). In addition, simultaneous tDCS-induced excitation over the primary motor cortex (leg area) and left temporal area contributed to improved overall athletic performance and decreased fatigue perception of professional bodybuilders (Kamali et al., 2019). Moreover, concurrent suppression of dorsolateral prefrontal cortex and stimulation of cerebellum by tDCS increased shooting scores of experienced pistol shooters (Kamali et al., 2019). The muscle strength parameters have also been found to be modulated through tDCS of the cerebellum (Kenville et al., 2020). Meta-analysis about the effect of anodal-tDCS on whole-body dynamic exercises (running and cycling) suggested that tDCS enhanced running and cycling time to exhaustion performance and the increased cortical excitability induced by tDCS might lead to lower ratings of perceived exertion (Shyamali Kaushalya et al., 2022).

Rowing has been one of the events of the modern Olympics since their reintroduction in 1896(Hosea and Hannafin, 2012). Competitive rowing is a high intensity demanding sport, requiring a high physical demand including highly developed aerobic and anaerobic capacity for optimal performance (Silva et al., 2021). The metabolic source of energy is predominantly aerobic during a rowing race and aerobic endurance is regarded as the most important physiological factor for successful rowing (Mikulic and Bralic, 2018). As rowing is a typical power endurance and whole-body activity requiring the activation of almost all muscles in the body, competition performance depends on factors such as physical fitness and power (both aerobic and anaerobic power), rowing techniques and tactics (Wei, 2021). The physiology of rowing is complex and the ability of the brain to maintain motor control and metabolism, with implication for cerebral blood flow, has been found to play an important role in the success of competitive rowing (Volianitis et al., 2020). Given the importance of brain for success in competitive events, there has been a rapidly increasing interest in exploring brain function and structure that associated with sport performance (Hill, 2020). In addition, the concept of “brain training and perceptual-cognitive training” including brain function, visual perception and decision-making, is proposed to improve athletic performance, and perceptual-cognitive process training has been applied to enhancing sport performance (Renshaw et al., 2018). Therefore, we speculated that measuring brain activity in athletes might be of great importance for understanding, predicting and monitoring athletic performance, and it might also be useful for the scientific selection of excellent athletes and develop appropriate sports training programs for athletes.

In the current study, we aimed to explore whether tDCS could improve the athletic performance of male rowing athletes and whether the improvements were related to the changes of brain activity and topological characteristics of functional brain network at rest. Therefore, resting-state functional magnetic resonance imaging (rs-fMRI) data of male rowing athletes were collected before and after tDCS over the left primary motor cortex while undergoing regular training. We hypothesized that tDCS intervention over the left primary motor cortex would increase brain activity and information transmission efficiency of the motor cortex, improving athletic performance of male rowing athletes. Based on this hypothesis, we compared differences of brain activity before and after tDCS by combining measures of amplitude of low-frequency fluctuation (ALFF) and regional homogeneity (ReHo), and obtaining information about the global and local efficiency of functional brain networks by graph theoretical analysis.

## Materials and methods

### Participants

The sample size estimation was performed based on results of previous related studies, in which the sample size ranged from 8 to 16. In this study, a power value (probability of correctly rejecting a false null hypothesis) of 0.8 was chosen given a type I error rate of α = 0.05, and the effect size was set to 0.4. Based on the above sample size calculation formula and parameters, the estimated minimum sample size to obtain sufficient test power was 10.

A total of 12 right-handed (confirmed using the Edinburgh handedness inventory) male rowing athletes who had at least 3 years of consistent rowing exercise were randomly chosen from those who volunteered to participate in the study. All participants were not informed about the specific purpose of the experiments and were all blinded for the stimulation intensity of tDCS. Participants were excluded with any neurological, psychiatric/psychological disorders or other severe somatic diseases, history of brain injury such as tumor or stroke, history of alcohol or substance dependence, and contraindications to MRI scanning. Table 1 summarized the demographic and information and athletic performance of male rowing athletes.

**Table 1:** Demographic characteristics and athletic performance of male rowing athletes between baseline and follow-up.

Characteristics	Baseline	Follow-up	Paired *t*	*p*-value
Gender (male/female)	12/0	-	-	-
Age (years)	16 ± 0	-	-	-
Educational level (years)	10.33 ± 0.49	-	-	-
BMI (kg/M^2^)	22.79 ± 2.53	-	-	-
LTP (watts)	212.50 ± 23.79	240.00 ± 14.77	−4.75	<0.01
M180LK (newton-meters)	133.75 ± 16.31	141.83 ± 16.97	−2.28	0.04
60LS (newton-meters)	112.17 ± 22.68	119.08 ± 23.19	−2.47	0.03

BMI, body mass index; LTP, lactate threshold power; M180LK: the average peak torque of left knee joint at 180°/s; 60LS: the highest peak torque of the left shoulder joint at 60°/s. Values of *p* were obtained by paired *t* tests. *p* < 0.05 indicated statistically significant differences.

**Figure 1:**
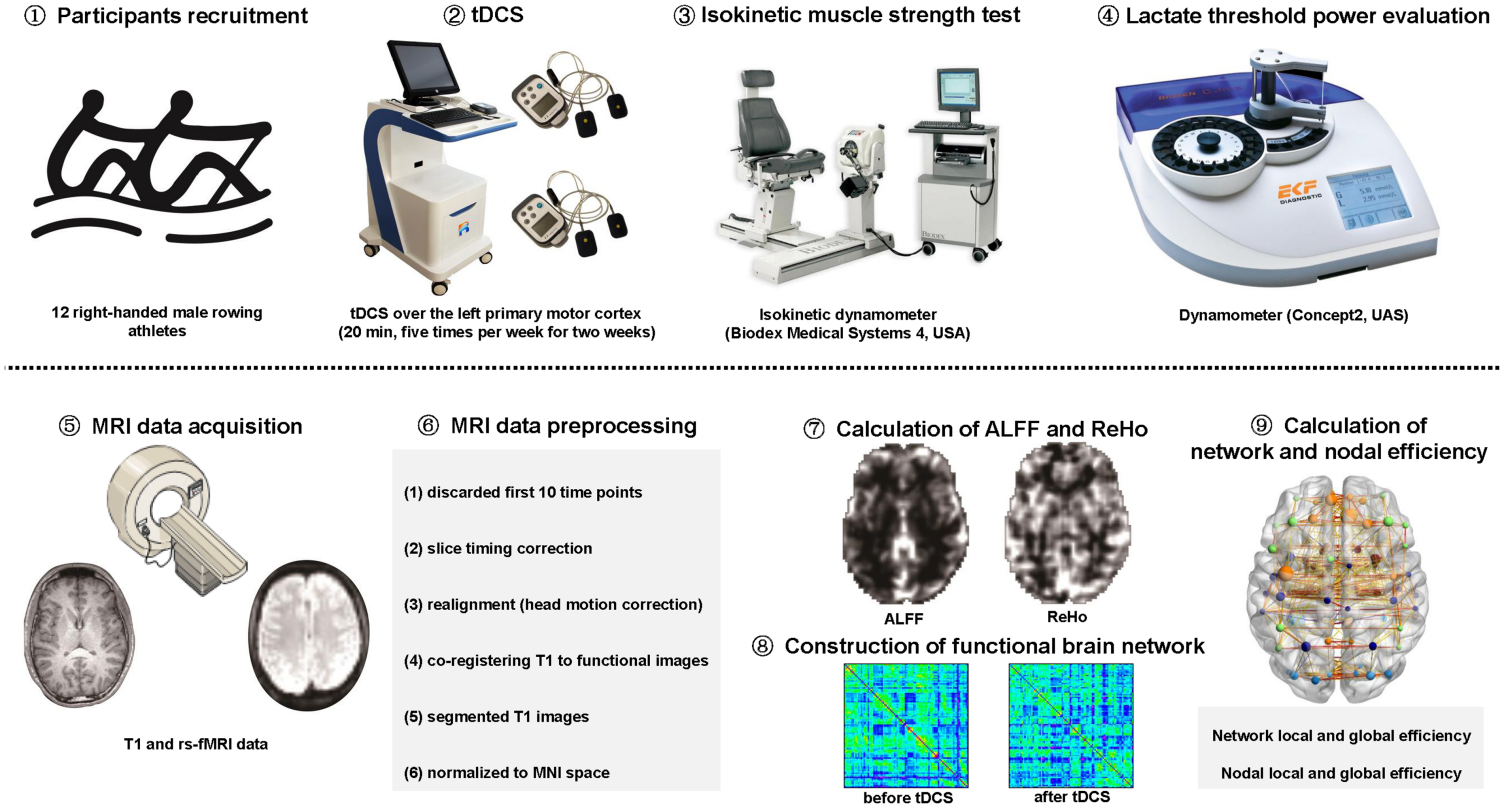
The schematic diagram for the study. tDCS: transcranial direct current stimulation. rs-fMRI: resting-state functional magnetic resonance imaging; MNI: Montreal Neurological Institute; ALFF: amplitude of low-frequency fluctuation; ReHo: regional homogeneity.

This study was approved by the ethics committee of Zhongda Hospital, School of Medicine, Southeast University. All participants were informed of the experimental procedure and signed informed consents before participating in this study. The study was carried out in accordance with the approved guidelines and regulations (Figure 1).

### Paradigm of tDCS

Twelve right-handed male professional rowing athletes were randomly and averagely divided into two groups. Both low-stimulation group (LSG, *n* = 6; 1 mA) and high-stimulation group (HSG, *n* = 6; 2 mA) received tDCS over the left primary motor cortex (20 min, five times per week for 2 weeks) while undergoing regular training. In previous studies, tDCS over left primary motor cortex had been proposed as a possible therapeutic technique for improving motor function (Bachmann et al., 2010; Hummel et al., 2010). In addition, tDCS over left primary motor cortex could exert an effect on adjacent brain regions and could lead a secondary effect on other distant regions in the brain (Lang et al., 2005). Therefore, tDCS over left primary motor cortex was effective at increasing the excitability of the dominant regions in the brain. In this study, the location of left primary motor cortex was determined as the C3 electrode according to the international 10/20 system (Beisteiner et al., 1995), as used in previous study (Lang et al., 2005).

### Isokinetic muscle strength test

In study, isokinetic dynamometer (Biodex Medical Systems 4, United States) was used to measure isokinetic muscle strength of the quadriceps and latissimus dorsi, which were essential for rowing. By the method of isokinetic dynamometry, the maximal strength of quadriceps and latissimus dorsi were measured by peak torque during the extended process of bilateral knee and shoulder joints at angular velocities of 60/s and 180/s. The highest peak torques, which considered as the index of explosive force, were recorded when male rowing athletes perform 5 slow traits at 60^°^/_s_ and 10 quick traits at 180^°^/_s_. M180LK and 60LS represented the mean peak torque of the left knee at 180^°^/_s_ and the highest peak torque of the left shoulder, at 60^°^/_s_, respectively.

### Lactate threshold and lactate threshold power evaluation

Lactate threshold (LT) is defined as the point at which lactate concentrations in the blood increase exponentially and the activities of the muscle become anaerobic during a graded incremental exercise test, which leading to muscle fatigue and shortness of breath. LT is usually determined as the corresponding power when the athlete’s blood lactate concentration reaches 4 mmol/L, which is called lactate threshold power (LTP). In this study, an indoor dynamometer (Concept2, UAS) was used to evaluate the power of pulling the oars with a distance of 1,000 m according to the method recommended by the International Rowing Federation (IRF). For the tests, the initial power was set at 170 watts and increased by 30 Watts per level with 30 s intermission. The blood acid level was tested with a portable blood lactic tester (EKF Blood Lactate, German) by collecting ear blood during every intermission. Finally, the curve of power-acid was calculated and LTP was acquired when the lactic level reached 4 mmol/l.

### MRI data acquisition and preprocessing

All male rowing athletes underwent MRI scans using a 3.0 Tesla Siemens MRI scanner at the Affiliated Nanjing Brain Hospital of Nanjing Medical University. The 3D T1-weighted images were acquired with the following parameters: repetition time (TR) = 1900 ms; echo time (TE) = 2.48 ms; flip angle (FA) = 9°; field of view (FOV) = 250 mm × 250 mm; acquisition matrix = 256 × 256; slice thickness = 1 mm; number of slices = 176. The rs-fMRI data were acquired with the following parameters: TR/TE = 2000/25 ms; FA = 90°; FOV = 240 mm × 240 mm; acquisition matrix = 64 × 64; slice thickness = 4 mm; number of slices = 36; number of volumes = 226.

MRI data of all participants were preprocessed by the software of Data Processing Assistant for Resting-State fMRI (DPARSF; Chao-Gan and Yu-Feng, 2010). The first 10 time points of each functional time series were discarded for the stabilization of the gradient magnetic field and the adaptation of participants to the scanning environment. The preprocessing procedure included the following steps: (1) slice timing correction; (2) realignment for head motion correction; (3) co-registering T1-weighted images to mean functional images; (4) segmented the co-registered T1 images into gray matter, white matter (WM) and cerebrospinal fluid (CSF); (5) normalized the functional images to the Montreal Neurological Institute (MNI) space with a resampling voxel size of 3 × 3 × 3 mm^3^.

### Calculation of ALFF and ReHo

ALFF: The calculation of ALFF was performed by the software of DPARSF (Chao-Gan and Yu-Feng, 2010). Firstly, the subsequent processing steps were conducted: (1) spatial smoothing with 4 mm × 4 mm × 4 mm full width at half-maximum (FWHM) Gaussian kernel; (2) removal of linear and quadratic trends, (3) regressing out nuisance signals such as those from WM and CSF, as well as global signals and motion parameters. Secondly, to obtain the power spectrum, the Fast Fourier Transform (FFT) was used to converted the time courses of all voxels into the frequency domain without band-pass filtering. Thirdly, the square root was calculated at each frequency of power spectrum. Finally, ALFF was calculated as the average square root of the power spectrum across 0.01–0.1 Hz, which was further divided by the global mean ALFF. Therefore, ALFF is a measure for exploring the level of regional spontaneous brain activity.

ReHo: The calculation of ReHo was also performed by the software of DPARSF (Chao-Gan and Yu-Feng, 2010). Firstly, the subsequent processing steps were conducted: (1) temporal band-pass filtering (0.01–0.1 Hz); (2) removal of linear and quadratic trends, (3) regressing out nuisance signals such as those from WM and CSF, as well as global signals and motion parameters. Secondly, ReHo maps were generated by calculating Kendall’s coefficient concordance (KCC, the correlations between the time series of a voxel and its nearest 26 neighbor voxels in a voxel-wise manner, also called ReHo value) of the time series of voxels with those of their nearest 26 neighbor voxels, which was further divided by the global mean KCC. Finally, to decrease spatial noise, spatial smoothing was applied with 4 mm × 4 mm × 4 mm FWHM Gaussian kernel to the ReHo maps, which were further standardized using *Fisher’s r*-to-*z* transformation. Therefore, ReHo is a voxel-based measure of brain activity that evaluates the degree of synchronization between the time series of a voxel and its nearest neighbors based on fluctuations of BOLD signals.

### Construction of functional brain network

Firstly, the automated anatomical labeling (AAL) template (Tzourio-Mazoyer et al., 2002) was used to parcellated the whole brain into 90 regions of interest (ROIs), which were considered as nodes of the functional brain network. Secondly, *Pearson’s* correlations between the time series of all ROIs, which defined as the strength of edges in the functional brain network, were extracted and transformed into z-scores using *Fisher’s r*-to-*z* transformation. Finally, a 90 × 90 correlation matrix was constructed for each participant.

### Calculation of network and nodal efficiency

The measures of network and nodal efficiency including local efficiency and global efficiency (Rubinov and Sporns, 2010), were calculated using the GRETNA toolbox (Wang et al., 2015). Given the absence of a gold standard for selecting a single threshold for the functional brain network, we thresholded the correlation matrix repeatedly over a series of sparsity threshold (0.01–1 with an interval of 0.01) and calculated the network and nodal efficiency including local efficiency and global efficiency of the resulting networks with different thresholds. In addition, area under the curve (AUC) of these measures were calculated within the whole range of sparsity thresholds (0.01–1). Global efficiency represents the capacity of parallel information transmission over the whole network while local efficiency represents the capacity of communication efficiency at the local level.

### Statistical analysis

The paired *t*-tests were applied to explore differences of athletic performance and AUC of network and nodal efficiency of male rowing athletes before and after tDCS using the Statistical Package for the Social Sciences (SPSS Inc., Chicago, IL, United States). *p* < 0.05 was considered statistically significant differences.

In addition, the paired *t*-tests were used to compare the differences of ReHo and ALFF values of male rowing athletes between baseline and follow-up using the REST Software (State Key Laboratory of Cognitive Neuroscience and Learning, Beijing Normal University, Beijing, China; Song et al., 2011). The significant differences were set at *p* < 0.005 (a minimum cluster size of 12 voxels, corrected for multiple comparisons at the cluster level by the AlphaSim program in REST software) for comparision of ReHo values while the significant differences were set at *p* < 0.05 (a minimum cluster size of 54 voxels, corrected for multiple comparisons at the cluster level by the AlphaSim program in REST software) for comparision of ALFF values.

## Results

### Demographic and athletic performance of male rowing athletes before and after tDCS

A total of 12 right-handed male rowing athletes [mean age (years): 16 ± 0, education level (years): 10.33 ± 0.49; BIM (kg/M^2^): 22.79 ± 2.53] were enrolled in the study (Table 1). Increased M180LK and 60LS were found in male rowing athletes after tDCS when compared with those before tDCS (Table 1). Improved athletic performance was found in male rowing athletes who received tDCS over the left primary motor cortex while undergoing regular training.

### Increased ALFF values of male rowing athletes after tDCS

**Figure 2:**
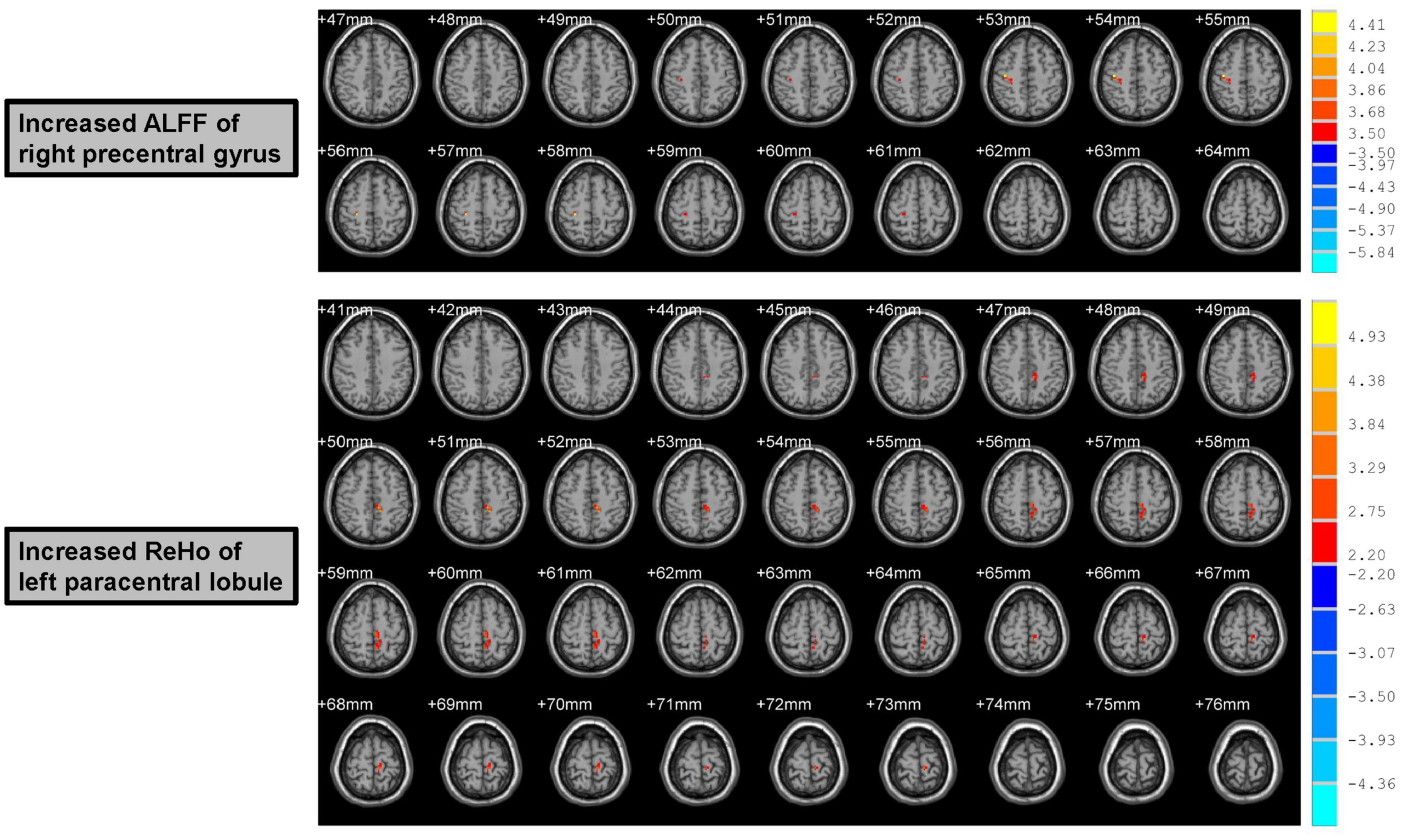
Increased ALFF and ReHo values of male rowing athletes after tDCS. ALFF, amplitude of low-frequency fluctuation; ReHo, regional homogeneity. tDCS, transcranial direct current stimulation. AAL, anatomic automatic labeling; MNI, Montreal Neurological Institute; *x*, *y*, and *z*: the coordinates of peak voxel of each cluster in the MNI space. For the measure of ALFF, the significant differences were set at *p* < 0.005 (a minimum cluster size of 12 voxels, corrected by the AlphaSim program in REST software). For the measure of ReHo, the significant differences were set at *p* < 0.05 (a minimum cluster size of 54 voxels, corrected by the AlphaSim program in REST software).

Increased ALFF values were found in the right precentral gyrus (Peak Montreal Neurological Institute (MNI) coordinates (x, y, z): 39, −18, 54; Number of voxels: 15; Peak *T* values: 4.45; *p* < 0.005, with a minimum cluster size of 12 voxels, corrected by the AlphaSim program in REST software) of male rowing athletes after tDCS when compared with those before tDCS (Table 2; Figure 2). Since the important role of the precentral gyrus in motor function, increased ALFF values in the precentral gyrus might indicate improved ability in initiating voluntary movement.

**Table 2:** Increased ALFF and ReHo values of male rowing athletes after tDCS.

Brain regions (AAL)	Peak MNI coordinates			Clusters	Peak *T* values	*p*-value
	*x*	*y*	*z*			
ALFF						
right precentral gyrus	39	−18	54	15	4.45	<0.005^a^
ReHo						
left paracentral lobule	−9	−33	51	76	5.47	<0.05^b^

ALFF, amplitude of low-frequency fluctuation; ReHo, regional homogeneity; tDCS, transcranial direct current stimulation; AAL, anatomic automatic labeling; MNI, Montreal Neurological Institute; *x*, *y*, and *z*: the coordinates of peak voxel of each cluster in the MNI space. ^a^ indicated that the significant differences were set at *p* < 0.005. (a minimum cluster size of 12 voxels, corrected by the AlphaSim program in REST software). ^b^ indicated that the significant differences were set at *p* < 0.05. (a minimum cluster size of 54 voxels, corrected by the AlphaSim program in REST software).

### Increased ReHo values of male rowing athletes after tDCS

Male rowing athletes showed increased ReHo values in the left paracentral lobule (Peak MNI coordinates (x, y, z): −9, −33, 51; Number of voxels: 76; Peak *T* values: 5.47; *p* < 0.05, with a minimum cluster size of 54 voxels, corrected by the AlphaSim program in REST software) following tDCS (Table 2; Figure 2). As the first somatosensory area and an important region connecting the precentral and postcentral gyrus (Lim et al., 1994; Zhang et al., 2016), increased ReHo values of the paracentral lobule implied higher function in somatic movement and sensation.

### Comparison of network efficiency of male rowing athletes before and after tDCS

**Figure 3:**
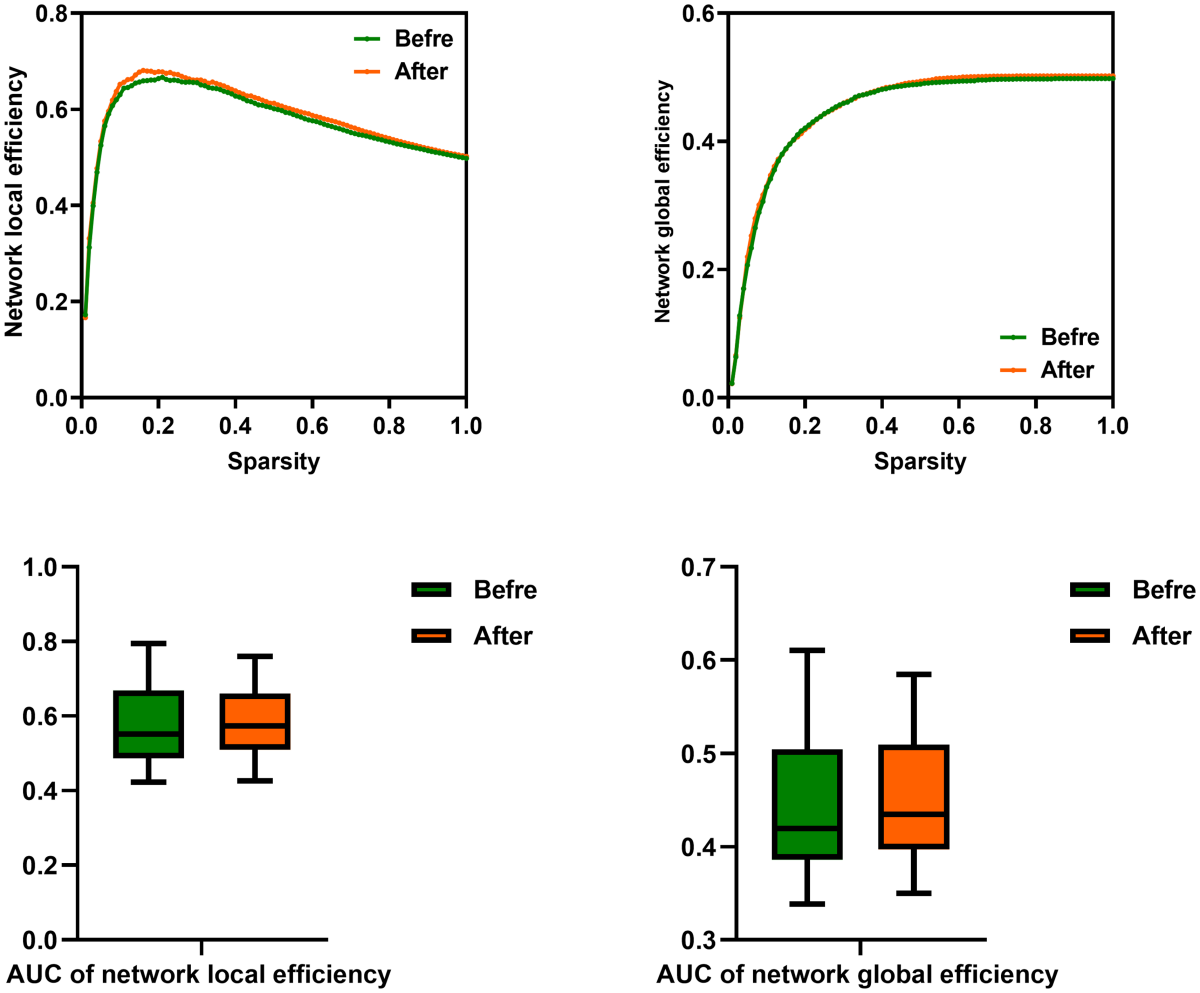
Comparison of network efficiency of male rowing athletes before and after tDCS. AUC, area under the curve; tDCS, transcranial direct current stimulation. Values of *p* were obtained by paired *t* tests. *p* < 0.05 indicated statistically significant differences.

In this study, the functional brain networks were thresholded with varied sparsity from 0.05 to 1 with step 0.01. No significant differences were found in the network local and global efficiency of functional brain network of male rowing athletes before and after tDCS (sparsity ranges: 0.05–1), as well as AUC of the network local and global efficiency (Table 3; Figure 3).

**Table 3:** Comparison of AUC of network and nodal efficiency of male rowing athletes before and after tDCS.

Characteristics	Baseline	Follow-up	Paired *t*	*p*-value
Network local efficiency	0.57 ± 0.11	0.58 ± 0.10	−0.21	0.84
Network global efficiency	0.44 ± 0.08	0.45 ± 0.07	−0.10	0.92
Nodal local efficiency	No brain regions showed significant differences			
Nodal global efficiency				
Left inferior frontal gyrus (opercular part)	0.35 ± 0.07	0.42 ± 0.08	−2.69	0.021

AUC, area under the curve; tDCS, transcranial direct current stimulation. Values of *p* were obtained by paired *t* tests. *p* < 0.05 indicated statistically significant differences.

### Comparison of nodal efficiency of male rowing athletes before and after tDCS

**Figure 4:**
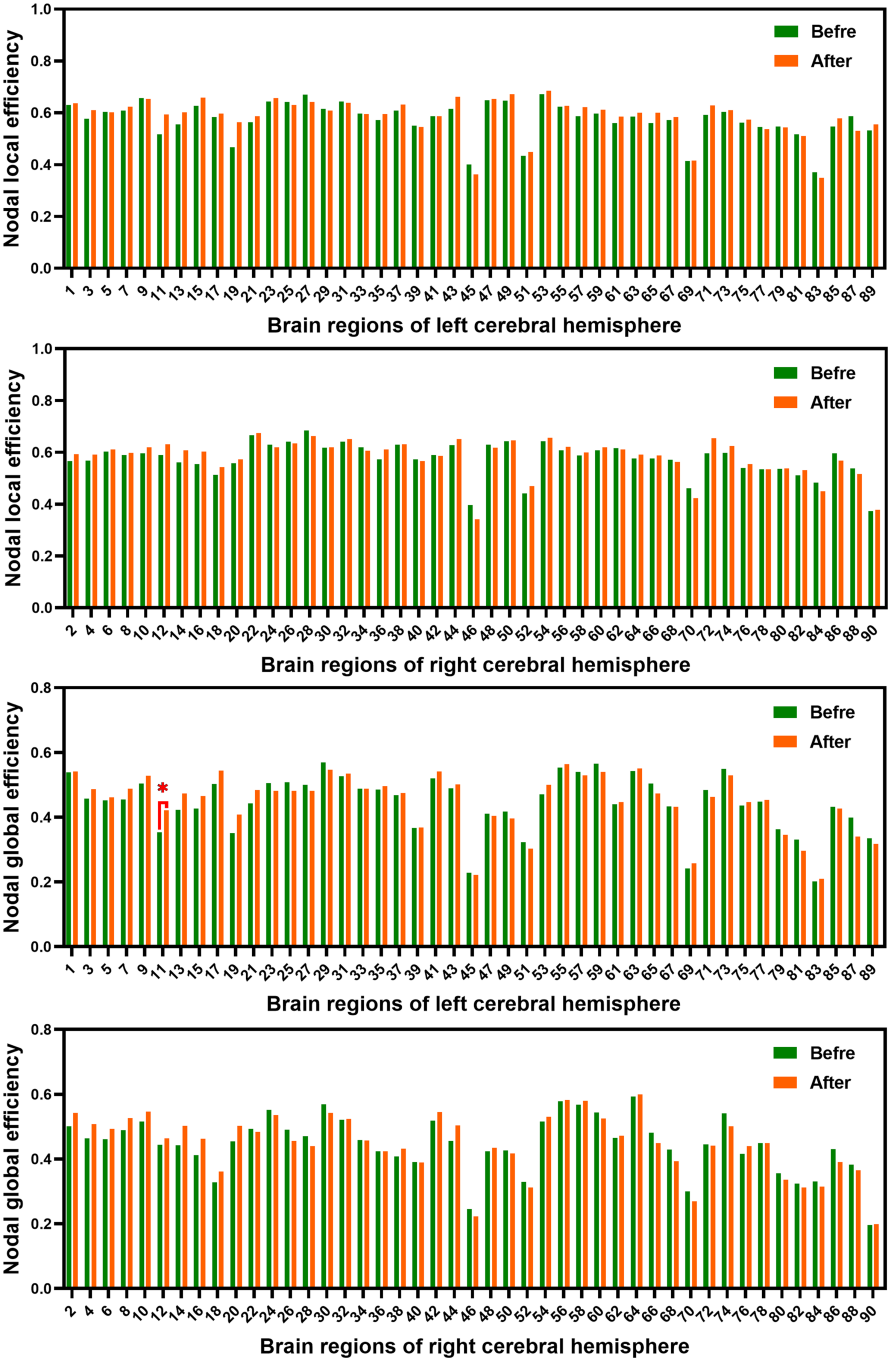
Comparison of AUC of nodal efficiency of male rowing athletes before and after tDCS. AUC, area under the curve; tDCS, transcranial direct current stimulation. Values of *p* were obtained by paired *t* tests. *p* < 0.05 indicated statistically significant differences.

**Figure 5:**
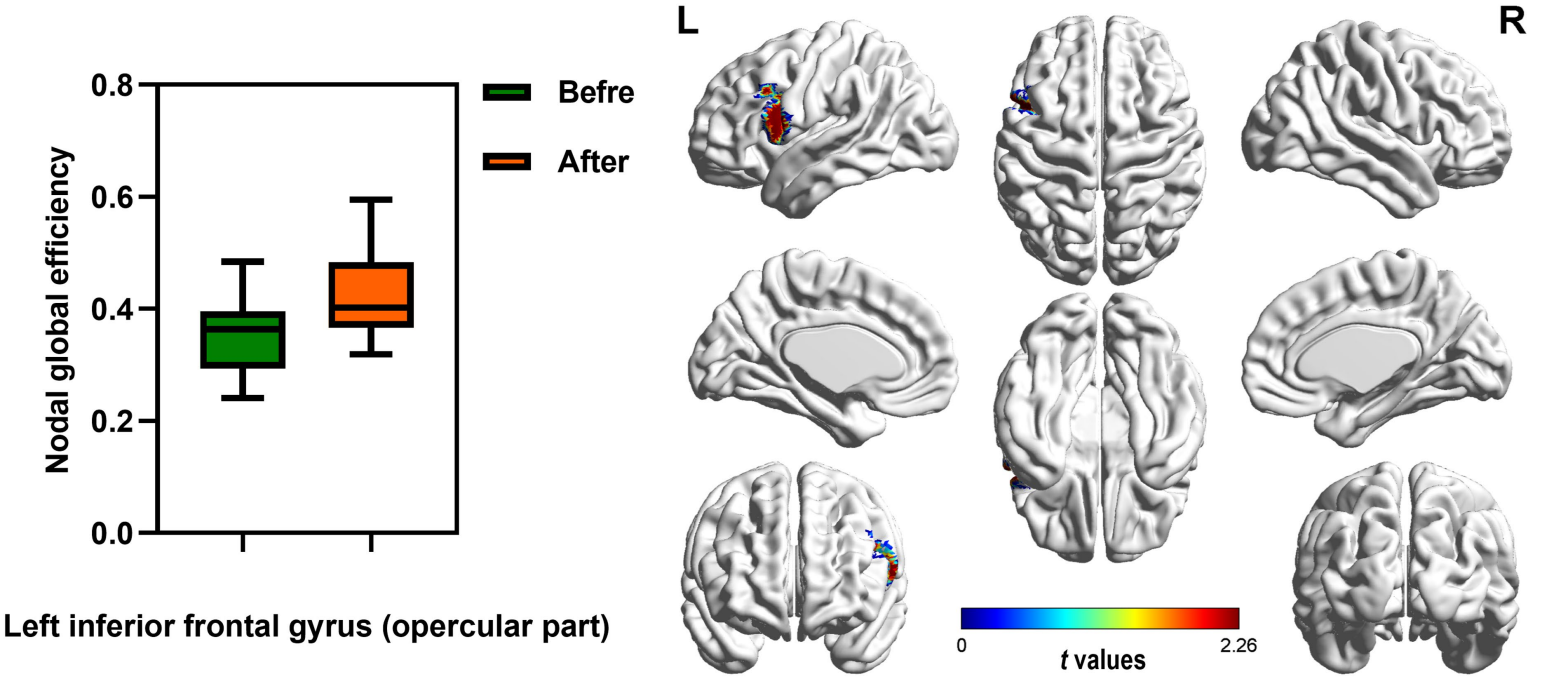
Brain region showed increased AUC of nodal global efficiency of male rowing athletes after tDCS. L: left cerebral hemisphere; R: right cerebral hemisphere. AUC, area under the curve; tDCS, transcranial direct current stimulation. Values of *p* were obtained by paired *t* tests. *p* < 0.05 indicated statistically significant differences.

In the range of 0.05–1, AUC of the nodal local and global efficiency in the functional brain networks for male rowing athletes before and after tDCS were calculated and then compared. No differences were found in AUC of the nodal local efficiency of male rowing athletes before and after tDCS (Table 3; Figure 4). However, increased AUC of nodal global efficiency was identified in the left inferior frontal gyrus (opercular part) of male rowing athletes after tDCS while no differences were found in AUC of the nodal global efficiency of other brain regions (Table 3; Figures 4, 5). As a key region in the cognitive-motor network, increased nodal global efficiency in the left inferior frontal gyrus (opercular part) might facilitate more efficient information transfer by greater functional connectivity with brain regions related to motor function in the brain.

## Discussion

To the best of our knowledge, the present study was the first to explore the central mechanisms of tDCS on improving the athletic performance of male rowing athletes from the perspectives of both regional brain activity and whole-brain functional network. In this study, we evaluated the effects of tDCS on the athletic performance of male rowing athletes and whether the effects were associated with the changes of the functional brain activity. We observed significant improvements in the athletic performance of male rowing athletes after tDCS over the left primary motor cortex while undergoing regular training. In addition, increased ALFF values in the right precentral gyrus and increased ReHo values in the left paracentral lobule, as well as increased nodal global efficiency in the left inferior frontal gyrus (opercular part), were found in male rowing athletes after tDCS. These findings suggested that simultaneous tDCS-induced excitation might potentially improve the overall athletic performance of male rowing athletes through increased regional spontaneous activity and capacity of parallel information transmission in brain regions related to motor function.

The technique of tDCS has been widely used in understanding brain function, the treatment of neuropsychiatric diseases and it has received increasing attention due to its potential impacts on brain activity in both healthy subjects and patients in the department of rehabilitation (Kuo et al., 2014; Ciullo et al., 2021). In addition, tDCS has also been used to enhance sport performance, facilitate neuroplasticity and training adaptations (Seidel and Ragert, 2019). The neuromodulatory effect of tDCS depends on the polarity: excitatory effect under the anodal electrode and inhibitory effect under the cathodal electrode, which is related to the shift of the resting membrane potential of the targeted neural cells (Stagg and Nitsche, 2011). With tDCS, a weak electric direct current is applied to the scalp with the intention to modulate the activity of a targeted brain area, which has been proven to induce positive effects on the physical performance of athletes (Grandperrin et al., 2020; Nakashima et al., 2021). In this study, we also observed significant improvements in the athletic performance of male rowing athletes received tDCS over the left primary motor cortex while undergoing regular training. Therefore, the potential central neural mechanisms underlining the effects of tDCS on athletic performance might be associated with the increased cortical excitability in the primary motor cortex. Evidence from neuroimaging studies showed that training-related increased activation was observed in the precentral gyrus and paracentral lobule while increased activation was found in the inferior frontal gyrus in individuals receiving real tDCS when compared to sham tDCS (Greenwood and Parasuraman, 2010; Wang et al., 2015). In addition, the precentral gyrus plays an important role in initiating voluntary movement while the inferior frontal gyrus, a key area of the cognitive-motor network, is vital for planning motor actions (Caspers et al., 2010; Rottensteiner et al., 2015). Therefore, we speculated that increased ALFF values in the right precentral gyrus and increased ReHo values in the left paracentral lobule might result from training while increased nodal global efficiency in the left inferior frontal gyrus (opercular part) might be caused by tDCS or tDCS coupled with training.

In this study, the results about the central mechanisms of tDCS on improving the athletic performance of male rowing athletes demonstrated increased ALFF values in the right precentral gyrus and increased ReHo values in the left paracentral lobule, as well as increased nodal global efficiency in the left inferior frontal gyrus (opercular part). Increased peak power output was found during maximal cycling incremental test following tDCS over the left temporal cortex, which could also modulate the excitability of the insular cortex and improve endurance performance (Okano et al., 2015). In addition, increased excitability in the primary motor cortex following tDCS administration was related to less input from other brain regions and induced the output required to recruit the muscle to produce a given force or power output (Angius et al., 2018). Increased inhibitory control and endurance cycling performance were found caused by changes in frontal lobe excitability was found in in healthy individuals following tDCS with the anodal electrode over the left dorsolateral prefrontal cortex (Angius et al., 2019). The tDCS over the left dorsolateral prefrontal cortex also increased the tolerance to the exercise performed with maximum load (Lattari et al., 2018). Therefore, the potential mechanism underlining the effects of anodal-tDCS on endurance motor performance might be also related with the increased cortical excitability in the primary motor cortex, which decreased the level of fatigue (Shyamali Kaushalya et al., 2022).

Given the complexity involved in exercise performance, there are multiple brain areas involved in exercise regulation, such as the prefrontal and parietal regions, anterior cingulate cortex, as well as some motor-related regions including precentral and paracentral lobule (Lutz, 2018). The precentral gyrus, which known as the primary motor cortex, plays an important role in motor control and the neurons in this brain region are involved in encoding distinct motor parameters, such as direction, velocity, position and muscle activity (Lillicrap and Scott, 2013). Higher functional connectivity was found between the left precentral gyrus and right postcentral and precentral gyrus in the endurance runners when compared with healthy controls (Cao et al., 2021). Considering the important role of the precentral gyrus in the motor control, increased ALFF values in the right precentral gyrus in this study might be associated with the improvements in the athletic performance of male rowing athletes who received tDCS over the left primary motor cortex. In addition, this result might indicate that tDCS over the left primary motor cortex could induce increased regional spontaneous activity of the right precentral gyrus, which were associated with better sensory-motor integration and interlimb coordination.

Being one part of both the prefrontal and parietal cortex, the paracentral lobule is a key region belonged to the motor-sensory network in the brain, which innervates motor and sensory modules of the contralateral extremities (Bruchhage et al., 2020; Deng et al., 2021). In order to achieve optimal athletic performance human brain has the ability to maintain sustained attention, multi-limb coordination and integrate signals from multiple sensory and motor regions in the brain (Bruchhage et al., 2020). In this study, increased ReHo values in the left paracentral lobule might be an adaptation to tDCS over the left primary motor cortex while undergoing regular training, which might lead to better temporal accuracy, spatial organization of movements and strong attentional control. In addition, increased fractal complexity of both precentral gyrus and paracentral lobules were found in observed in elite basketball players, which were related to the competence required to play basketball (Kim et al., 2022).

The inferior frontal gyrus related to the action observation and imitation, which connected with supplementary motor area, is considered as a vital area for planning and initiating motor actions, and is a key region in the cognitive-motor network of brain (Caspers et al., 2010; Rottensteiner et al., 2015). Stronger effects were found in the left inferior frontal gyrus and precentral gyrus in motor experts when compared with novices, which suggested that these two regions were parts of the mirror neuron system and were more involved in understanding others’ action goals after long-term motor training (Yang, 2015). This finding verified the idea that executive control network was the key structure for converting selective and spatial attention in athletes with high motor expertise (Wu et al., 2007). Moreover, the inferior frontal gyrus was found to be of special importance for inhibitory control of motor responses and impaired network integrity of the inferior frontal gyrus was related to vigorous physical activity (Kullmann et al., 2014). Therefore, in study, increased nodal global efficiency in the left inferior frontal gyrus (opercular part), which had been shown to be crucial in the control of body movements (Kullmann et al., 2014; Suss et al., 2022), might facilitate more efficient information transfer by greater functional connectivity with brain regions related to motor function in the brain (such as other areas of the frontal lobe, precentral and postcentral gyrus, somatosensory cortex) of male rowing athletes received tDCS.

In the present study, rowing is a bimanual task. Increased ALFF values in the right precentral gyrus and increased ReHo values in the left paracentral lobule were found in male rowing athletes following tDCS. Moreover, increased nodal global efficiency was identified in the left inferior frontal gyrus (opercular part) of male rowing athletes after tDCS. We hypothesized that the existence of a rather widely distributed motor network was responsible for bimanual task. The increased activities in the left paracentral lobule and inferior frontal gyrus (opercular part) might be resulted from the treatment of tDCS. The activation in the right precentral gyrus suggested that this region was also responsible for the control of bimanual motor actions, which might be the result of training. However, in light of differences in the relative balance of left and right M1 in the context of unimanual versus bimanual tasks, it was difficult to interpret the different changes in left and right M1 in a bimanual task. Therefore, both unimanual and bimanual tasks should be performed in our further studies, to explore the detailed mechanisms underlying different changes in left and right M1 in athletes after tDCS.

However, there were several limitations in this study. Firstly, the sample size of this study was relatively small for exploring the central mechanisms of tDCS on improving the athletic performance, although some previous studies used similar sample sizes. Secondly, we could not determine whether these functional alterations were parts of both rowing training and tDCS or a consequence of only tDCS because of the paradigm of tDCS (rowing athletes received tDCS over the left primary motor cortex while undergoing regular training). In addition, the lack of a tDCS control or sham group might limit the interpretation of the findings and might be a source of bias in this study. Finally, the effects of tDCS on athletic performance and brain activity of male rowing athletes needed to be verified by personalized stimulation parameters since individual differences in physiological parameters might lead to heterogeneous stimulation responsivity. Therefore, further studies would be conducted between group receiving tDCS (low-stimulation and high-stimulation, with larger sample size and personalized stimulation parameters) and sham group with and without training with personalized stimulation parameters.

## Conclusion

Taken together, our findings provided novel evidences that tDCS over the primary motor cortex could enhance the athletic performance of male rowing athletes through the right precentral gyrus and left paracentral lobule, as well as left inferior frontal gyrus, which were important motor regions and network of the brain. This was the first study to provide brain stimulation protocols to enhance regional spontaneous activity and capacity of parallel information transmission in brain regions related to motor function and improve subsequently the athletic performance. Given the positive effects of tDCS coupled with physical exercise on athletic performance and muscular strength (the rapid generation of peak muscular force), it could be considered as a supportive training tool that allowing to enhance training effectiveness for rowing athletes.

## Data availability statement

The raw data supporting the conclusions of this article will be made available by the authors, without undue reservation.

## Ethics statement

The studies involving human participants were reviewed and approved by the ethics committee of Zhongda Hospital, School of Medicine, Southeast University. Written informed consent to participate in this study was provided by the participants’ legal guardian/next of kin.

## Author contributions

MM, YY, XY, JG, and ZH designed the experiments. MM, XY, JG, YZ, and ZH contributed to clinical data collection and assessment. MM, WZ, JC, YF, ZX, and YX analyzed the results. MM, WZ, JC, and YF wrote the manuscript. MM, JC, and YF approved the final manuscript. All authors contributed to the article and approved the submitted version.

## Funding

The work was supported by the grants of: Jiangsu Provincial Sports Bureau (no. ST191101) and Nanjing Medical Science and technology development Foundation (no. YKK19163).

## Conflict of interest

The authors declare that the research was conducted in the absence of any commercial or financial relationships that could be construed as a potential conflict of interest.

## Publisher’s note

All claims expressed in this article are solely those of the authors and do not necessarily represent those of their affiliated organizations, or those of the publisher, the editors and the reviewers. Any product that may be evaluated in this article, or claim that may be made by its manufacturer, is not guaranteed or endorsed by the publisher.
